# Broad HIV-1 inhibition *in vitro* by vaccine-elicited CD8^+^ T cells in African adults

**DOI:** 10.1038/mtm.2016.61

**Published:** 2016-08-31

**Authors:** Gaudensia Mutua, Bashir Farah, Robert Langat, Jackton Indangasi, Simon Ogola, Brian Onsembe, Jakub T Kopycinski, Peter Hayes, Nicola J Borthwick, Ambreen Ashraf, Len Dally, Burc Barin, Annika Tillander, Jill Gilmour, Jan De Bont, Alison Crook, Drew Hannaman, Josephine H Cox, Omu Anzala, Patricia E Fast, Marie Reilly, Kundai Chinyenze, Walter Jaoko, Tomáš Hanke, the HIV-CORE 004 study group

**Affiliations:** 1 KAVI-Institute of Clinical Research, University of Nairobi, Kenya;; 2 Human Immunology Laboratory, International AIDS Vaccine Initiative, Imperial College, London, UK; 3 Jenner Institute, University of Oxford, Oxford, UK; 4 Emmes Corporation, Rockville, Maryland, USA; 5 International AIDS Vaccine Initiative-New York, New York, New York, USA; 6 ICHOR Medical Systems, Inc., San Diego, California, USA; 7 Karolinska Institute, Stockholm, Sweden; 8 International Research Center for Medical Sciences, Kumamoto University, Japan; 9

## Abstract

We are developing a pan-clade HIV-1 T-cell vaccine HIVconsv, which could complement Env vaccines for prophylaxis and be a key to HIV cure. Our strategy focuses vaccine-elicited effector T-cells on functionally and structurally conserved regions (not full-length proteins and not only epitopes) of the HIV-1 proteome, which are common to most global variants and which, if mutated, cause a replicative fitness loss. Our first clinical trial in low risk HIV-1-negative adults in Oxford demonstrated the principle that naturally mostly subdominant epitopes, when taken out of the context of full-length proteins/virus and delivered by potent regimens involving combinations of simian adenovirus and poxvirus modified vaccinia virus Ankara, can induce robust CD8^+^ T cells of broad specificities and functions capable of inhibiting *in vitro* HIV-1 replication. Here and for the first time, we tested this strategy in low risk HIV-1-negative adults in Africa. We showed that the vaccines were well tolerated and induced high frequencies of broadly HIVconsv-specific plurifunctional T cells, which inhibited *in vitro* viruses from four major clades A, B, C, and D. Because sub-Saharan Africa is globally the region most affected by HIV-1/AIDS, trial HIV-CORE 004 represents an important stage in the path toward efficacy evaluation of this highly rational and promising vaccine strategy.

Despite remarkable progress in decreasing human immunodeficiency virus type 1 (HIV-1) transmission and AIDS-related deaths by antiretroviral drugs,^1^ an effective, prophylactic HIV-1 vaccine will be the best strategy for realistically ending the AIDS epidemic. For the most efficient control of HIV-1, a vaccine will likely have to induce both functional binding or broadly neutralizing antibodies (bnAbs) and effective cytotoxic CD8^+^ T cells.^2^ While induction of appropriate B-cells to produce bnAbs currently holds promise, CD8^+^ T cells are important to limit and remove HIV-1-infected cells.^3,4^ Broadly specific CD8^+^ T cells of a noncanonical type (restricted by Mamu tissue antigens of classes Ib/E and II) were associated with control and clearance of pathogenic simian immunodeficiency virus infection in 54% of about 100 experimentally challenged rhesus macaques.^5–8^ In humans, the first appearance of human leukocytes antigen (HLA) class Ia-restricted CD8^+^ T cells forces extensive virus escape in targeted epitopes during acute HIV-1 infection^9,10^ and correlates with a decrease in acute viremia,^10^ however, T cells eventually fail to prevent AIDS.^3^ Also genome-wide association studies showed protective effects of certain HLA class I allotypes.^11^ Our aim is to understand and induce protective T-cell responses, which will complement vaccine-elicited binding or broadly neutralizing antibodies in prevention as well as assist HIV-1 cure.

Functional correlates of T-cell control of HIV-1 replication are likely to be a combination of several qualities, many of which are critically important. Thus, in addition to the efficient recognition of peptide-loaded HLA molecules,^12^ rapid expansion following exposure to cognate antigens,^13,14^ efficient killing of infected cells,^13–15^ production of soluble antiviral factors^13,14,16^ and the use of shared T-cell receptors (public clonotypes),^17^ we believe CD8^+^ T-cell specificity^18–24^ and breadth^2,25,26^ of epitope recognition are key to a successful control of extremely variable pathogens such as HIV-1. The most relevant evaluation of the CD8^+^ T-cell effector functionality prior to efficacy trials in humans is the *in vitro* viral inhibition assay (VIA).^27–36^ VIA collectively measures T-cell functions by quantifying reduction in HIV-1 replication in cultured autologous CD4^+^ T cells, and does so in the context of immune response-evasive mechanisms.^36^ Furthermore, VIA permits functional identification of inhibitory epitopes^27^ and the use of a number of HIV-1 isolates, including transmitted/founder viruses, to assess the breadth of the T-cell response inhibition over diverse HIV-1 isolates.^27–31,34^

The biggest roadblock for successful vaccine development remains the HIV-1 genetic variability, which has diversified circulating isolates globally and allows HIV-1 to change and escape immune attacks. There are several vaccine strategies that address HIV-1 variability,^22,24,37–39^ ours focuses the immune responses on the most conserved regions of the HIV-1 proteome.^20,21^ Functionally and structurally conserved protein regions are common to most global variants, responses to them are associated with a better efficiency of HIV-1 control^40–42^ and mutations in them typically lead to a replicative fitness loss.^43–46^ These subprotein regions are epitope rich and contain many subdominant epitopes.^47–51^ In contrast, full-length proteins, even the most conserved such as Gag,^52,53^ inevitably include hypervariable regions that interfere with responses to conserved epitopes, while a string of known conserved 9-mer epitopes^22,24^ is too narrow in breadth to adequately cover the complexity of the global potential T-cell epitopes and its design is dependent on current and likely biased knowledge of already identified epitopes.^20,21,54^ The first generation conserved region T-cell immunogen HIVconsv utilized alternating clade consensus sequences.^21^ In trial HIV-CORE (*CO*nserved *RE*gion) 002 in HIV-1-negative adults in Oxford, the HIVconsv immunogen proved the concept that conserved regions, when taken out of the context of full-length proteins/virus and delivered by a potent combination of simian (chimpanzee) adenovirus ChAdV-63 and poxvirus modified vaccinia virus Ankara (MVA), can induce robust CD8^+^ T-cell responses to many normally subdominant epitopes. These HIVconsv-specific T cells were induced in 26/26 (100%) of vaccine recipients. For two regimens, these reached a median of over 5,000 spot-forming units (SFU)/10^6^ of peripheral blood mononuclear cells (PBMCs), and recognized a median of 10 CD8^+^ and CD4^+^ T-cell epitopes.^28^ The CD8^+^ cells could be stimulated to proliferate and inhibit HIV-1 replication of several HIV-1 isolates *in vitro* in VIA.^27,28^ Here, we aimed to test the conserved-region T-cell vaccine strategy in HIV-1-negative adults in Nairobi, a genetically and environmentally highly relevant population for the eventual deployment of an effective HIV-1-vaccine, and to compare safety and immunogenicity of the vaccine regimens to those obtained in HIV-1-uninfected Oxford adults. These results are on the critical developmental path of the conserved-region T-cell platform toward a phase 2b efficacy evaluation in this region of the greatly improved second generation bivalent mosaic tHIVconsvX immunogens.^39^

## Results

### Vaccines, regimens, and study population of the HIV-CORE 004 trial

**Figure 1:**
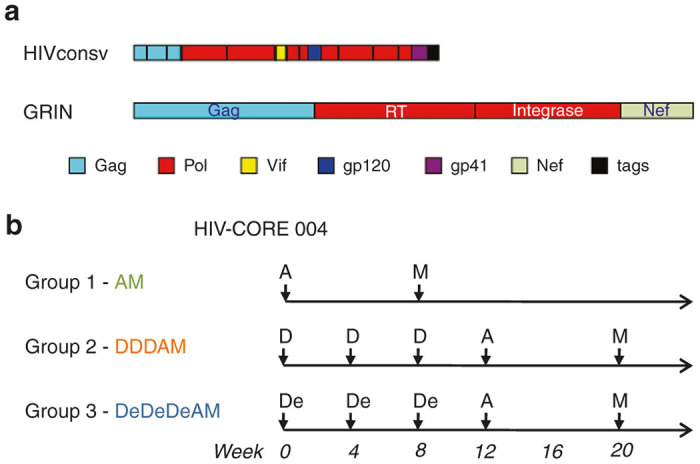
Trial HIV-CORE 004 immunogens and Group regimens. (**a**) Schematic representations of the two vaccine immunogens delivered by the trial vaccines. HIVconsv is assembled from 14 highly functionally conserved regions of HIV-1 proteins using alternating clade A, B, C, and D consensus sequences and delivered by pSG2 DNA and nonreplicating poxvirus modified vaccinia virus Ankara (MVA). GRIN is a fusion of full-length Gag, Reverse Transcriptase, Integrase, and Nef proteins of clade A, delivered by nonreplicating human adenovirus serotype 35. The HIVconsv protein was 776 aa long, of which 604 aa (78%) were contained in GRIN; these 604 aa of GRIN had 97.6% homology with the HIVconsv immunogen (Supplementary Figure S1). Individual regions/proteins are colour-coded and drawn relatively to scale. (**b**) Trial recruits were randomized into vaccine or placebo at a ratio of 20:4 per each regimen Group. D and De - pSG2.HIVconsv DNA without or with electroporation, respectively; A - Ad35-GRIN; and M - MVA.HIVconsv.

Immunogen HIVconsv is assembled from 14 highly conserved subprotein regions of HIV-1 using alternating clade consensus sequences (Figure 1a). It was highly immunogenic in HIV-negative adults in Oxford when delivered by a combination of plasmid pSG2 DNA (D), nonreplicating chimpanzee adenovirus ChAdV-63 (C) and nonreplicating MVA (M).^28^ Based on the peak IFN-γ ELISPOT assay frequencies of vaccine-elicited T cells, the three heterologous prime-boost regimens tested in Oxford ranked in the relative order of potency of DDDCM≥CM>>DDDMC. Here, we aimed at repeating the two most immunogenic regimens in the South and assessing whether the CM regimen benefits significantly from the DDD prime. In addition, we also further enhanced the DNA prime by electroporation using the TriGrid Delivery System of Ichor Medical Systems designed for intramuscular (i.m.) administration of DNA (De).^55^ Upon losing access to the ChAdV-63 vector, we substituted the ChAdV63.HIVconsv vaccine component with Ad35-GRIN (A), whereby nonreplicating human adenovirus 35 expresses a fusion protein of HIV-1 Gag-Reverse Transcriptase-Integrase-Nef of HIV-1 clade A (Figure 1a).^55,56^ The chimeric HIVconsv protein has 776 amino acids (aa) in length, of which 604 aa (78%) are contained in GRIN; within these 604 aa, there are 14 aa substitutions giving a 97.6% homology with the HIVconsv immunogen (Supplementary Figure S1). This made the HIVconsv and GRIN vaccines sufficiently similar to be combined in a prime-boost regimen (Figure 1b) and address our trial goals.

**Figure 2:**
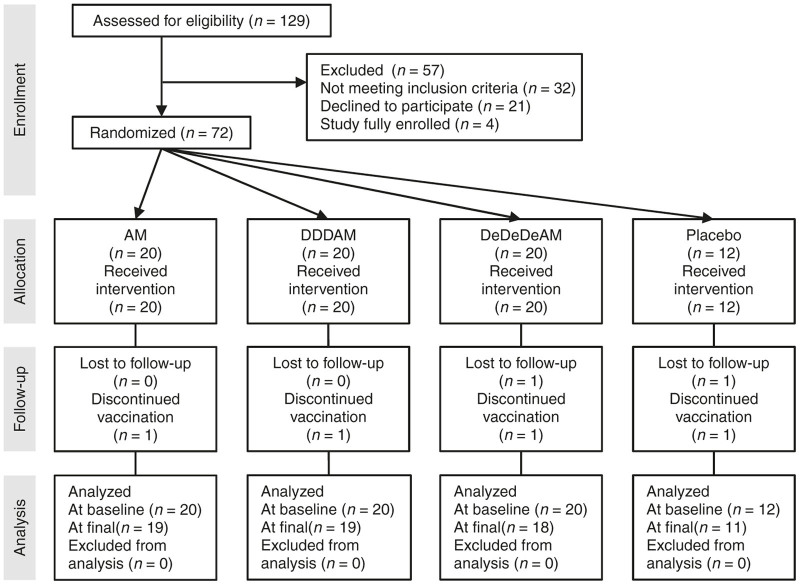
CONSORT flow diagram for HIV-CORE 004. Among the 60 volunteers allocated to the vaccine, three discontinued vaccinations: one volunteer refusal (after receipt of the first vaccination in Group AM) led to study termination, while one volunteer with pregnancy (after receipt of the third vaccination in Group DeDeDeAM) and one volunteer with an adverse event (after receipt of the fourth vaccination in Group DDDAM) completed their nonvaccine visits for the study. All volunteers were included in the safety analysis. For the three vaccine recipients who discontinued vaccinations, immunogenicity data were censored after the time of the missed vaccination. One placebo recipient was lost to follow-up after receipt of the fourth vaccination.

In trial HIV-CORE 004, 72 HIV-negative adults were recruited in the Kangemi district of Nairobi, Kenya, and randomized to receive either vaccine or placebo regimens at a ratio of 20 vaccine: 4 placebo (Figure 2). Volunteers in Group AM received 5 × 10^10^ virus particles (vp) of Ad35-GRIN or placebo i.m. at baseline followed by 2 × 10^8^ PFU of MVA.HIVconsv or placebo i.m. at 8 weeks. Volunteers in Group DDDAM and Group DeDeDeAM received three 4-mg doses of pSG2.HIVconsv DNA without (D) or with electroporation (De) or placebo i.m. at 0, 4, and 8 weeks followed by Ad35-GRIN and MVA.HIVconsv or placebo i.m. at weeks 12 and 20, respectively (Figure 1b). Each dose of vaccine and placebo was delivered by two injections, one into each arm. All volunteers were tissue typed (Supplementary Table S1). Median volunteer age was 27.5 (range 18–49) years, 57% were females and all were black Africans (Table 1). Fifty five volunteers reported social benefit from disclosure of study participation, while only five volunteers reported social harm.

### All regimens of experimental vaccines had good safety profiles

**Figure 3:**
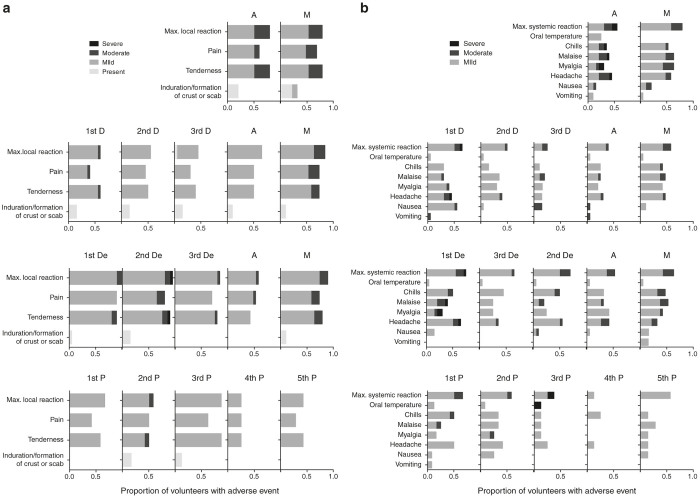
Maximum severity of adverse events. Local (**a**) and systemic (**b**) solicited adverse events over 7 days. The bars are colour-coded as indicated according to the severity of the adverse events in the corresponding fraction of volunteers.

Overall, the tested vaccines including the electroporation procedure were well tolerated with similar safety profiles in all three groups (Figure 3). No vaccine-related serious adverse events occurred in any of the groups. A total of 239 adverse events were reported in 21 weeks, of which the majority (69%) were of mild or moderate severity and 96% were considered unrelated to the vaccines by the clinical investigator. Five volunteers reported severe (Grade 3) reactogenicity events including malaise, chills, myalgia, headache, and tenderness at the vaccination site and elevated oral temperature, which resolved within 2 days. Two volunteers developed severe (Grade 3) reactogenicity events after receiving the Ad35-GRIN vaccination. Two volunteers: one in Group DDDAM and another in Group DeDeDeAM developed severe (Grade 3) reactogenicity events after receiving the first pSG2.HIVconsv DNA vaccination. One volunteer in the placebo group reported severe Grade 3 fever after the third vaccination. Only one volunteer reported severe tenderness (graded 3 on a scale of 1–4) 30 minutes after DNA electroporation. There were two individuals with clinically significant reported laboratory abnormalities in Group DDDAM. One male volunteer had Grade 3 alanine transaminase and Grade 4 aspartate transaminase elevations (on a scale of 1–4) 6 months after the last vaccination. This was considered likely related to self-reported alcohol intake prior the clinic visit and the liver function in this volunteer resolved. Another volunteer had Grade 3 neutropenia at the time of his fourth vaccination, which was judged as unlikely related to the vaccine. Further vaccination was discontinued and repeat hematology tests confirmed neutropenia ranging from Grade 2 value of 0.93 × 10^3^ cells/µl to Grade 3 values of 0.59 × 10^3^ cells/µl. Otherwise the volunteer remained healthy until the end of the trial. Two pregnancies and no HIV-1 acquisition were reported during the study. Volunteers have been enrolled in a long-term follow-up study.

### All regimens induced high frequencies of T cells against conserved regions of HIV-1

**Figure 4:**
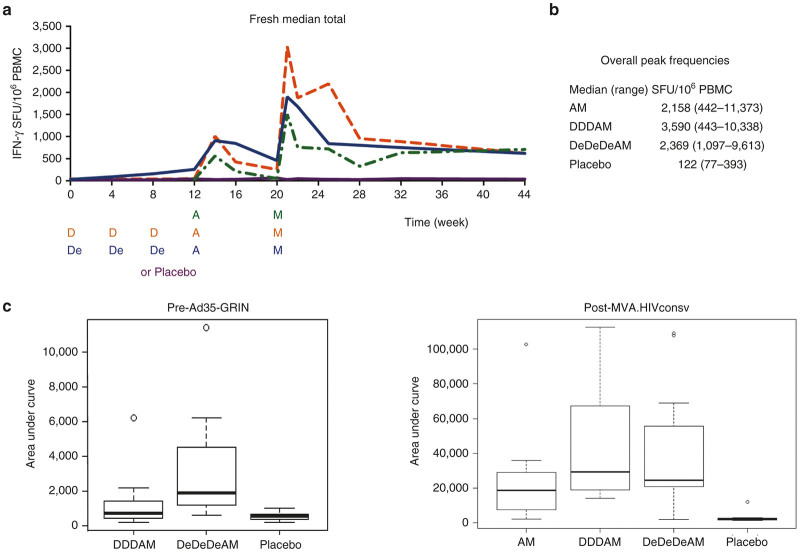
Frequencies of interferon (IFN)-γ-producing T cells recognizing conserved regions of HIV-1 induced by vaccination in African adults. Freshly isolated *ex vivo* peripheral blood mononuclear cells (PBMCs) were used in an IFN-γ ELISPOT assay using 199 15/11 peptides spanning the full-length of the HIVconsv immunogen arranged into six pools P1-P6 to assess the frequencies of vaccine-elicited T cells. (**a**) The diagram shows the kinetics of the vaccine-induced responses. The vaccine/placebo administration time points are indicated below the graph; note that for the AM regimen (dashed green line), the whole curve was shifted by 12 weeks to visualize comparison of the AM, DDDAM, and DeDeDeAM regimens, *i.e.*, A and M or placebo were administered at weeks 0 and 8, but are depicted at weeks 12 and 20, respectively. The data are presented as medians of total (sum of P1–P6) net frequencies for each group/regimen at the indicated time points. Responses for each individual separately can be seen in Supplementary Figure S2. Part (**b**) summarizes the overall peak frequencies of HIVconsv-specific T cells detected for individual volunteers. (**c**) Area under curve (AUC) analysis assessing the differences between regimens. AUC was calculated for individual volunteers (14 AM, 15 DDDAM, 17 DeDeDeAM and 8 Placebo) with complete number of visits over the compared study periods: time from baseline to the time of the administration of Ad35-GRIN (week 0–12) for direct comparison of the electroporation on the induction of T cells by pSG2.HIVconsv DNA (left) and from the administration of the last vaccine modified vaccinia virus Ankara (MVA).HIVconsv until the end of the study (week 20–44) (right). The graphs show a median with boxed interquartile range. Using the Wilcoxon rank sum test for comparison of regimens, the *P*-values before correcting for five comparisons were as follows: DDD versus DeDeDe − *P* = 0.0002; DDDAM versus DeDeDeAM − *P* = 0.9; AM versus DDDAM − *P* = 0.07; AM versus DeDeDeAM − *P* = 0.08; and AM versus DDDAM+DeDeDeAM − *P* = 0.04.

*Ex vivo* IFN-γ ELISPOT assay in freshly isolated PBMCs was employed for enumeration of HIVconsv-specific T cells induced by the three vaccine regimens. One hundred and ninety nine 15-mer peptides overlapping by 11 aa (15/11) spanning the entire HIVconsv protein were arranged into six peptide pools P1–P6 as described previously^28^ and used to determine the overall magnitude (using the sum of the six responses) and breadth (number of pools responding) of T-cell responses. Figure 4a shows the group kinetics of the elicited responses throughout the vaccination and follow-up periods. Our subsequent ELISPOT assay analyses focused on volunteers’ peak responses and a measure of total response was estimated by the area under curve (AUC). The peak response was chosen for analysis because vaccine-elicited responses did not peak at the same time point after vaccine administrations for all volunteers (Supplementary Figure S2) and peak expansions of T-cell populations correlate well with the size of the subsequent long-lived T-cell memory.^57^ All vaccine recipients (100%) responded to the vaccination, as shown by HIVconsv-specific T-cells capable of producing IFN-γ upon *in vitro* peptide restimulation. All regimens induced high frequencies of HIVconsv-specific T cells in circulating PBMCs, which per volunteer peaked at a median (range) of 2,158 (442–11,373), 3,590 (443–10,338), and 2,369 (1,097–9,613) SFU/10^6^ PBMC for the AM, DDDAM, and DeDeDeAM regimens, respectively (Figure 4b). These median peak frequencies of the three regimens were not statistically different from each other and no significant differences among the three regimens in HIVconsv-specific IFN-γ-producing cells were detected 24 weeks after the last MVA.HIVconsv (week 44). The median (range) of the AUC values for the AM, DDDAM, and DeDeDeAM regimens between week 20 and 44 were 18,548 (2,148–102,712), 29,198 (14,876–112,613), and 24,499 (1,796–189,146), respectively. While individual groups were not statistically separable, comparing AM to the combined DDDAM and DeDeDeAM regimens suggested a statistically significant difference (*P* = 0.04) before correction for multiple comparisons (Figure 4c). Overall, while the three tested regimens were inseparable by the frequencies of vaccine-generated IFN-γ-producing memory T cells at peaks and at the end of the study, the kinetics and AUC analysis of the vaccine-elicited T-cell frequencies suggested some subtle differences resulting from the DNA prime.

### Electroporation enhanced responses to i.m. DNA administration

Consistent with our previous observations in humans,^28,58,59^ a straight i.m. needle injection of 4 mg of “naked” pSG2.HIVconsv DNA alone elicited marginal responses that peaked with median (range) of 81 (20–1,410) SFU/10^6^ PBMCs. Electroporation immediately following i.m. injection of DNA increased the peak frequencies to 393 (123–1,391) SFU/10^6^ PBMCs (peak DDD versus peak DeDeDe: *P* = 0.004) and a significant enhancement of T-cell frequencies induced by DNA over the first 12 weeks of the protocol was also detected by the AUC analysis over weeks 0–12 (DDD versus DeDeDe: *P* = 0.0002) (Figure 4c). With respect to priming, nonelectroporated and electroporated DNA both showed a significant increase in Ad35-GRIN-elicited peak responses of 585 (76–4,225) SFU/10^6^ PBMCs after A alone to 1,433 (153–3,458) SFU/10^6^ PBMCs after DDDA (peak A versus peak DDDA: *P* = 0.01) and 1,155 (73–2,962) SFU/10^6^ PBMCs after DeDeDeA (peak A versus peak DeDeDeA: *P* = 0.03).

### Vaccine-elicited T-cell responses recognized multiple HIVconsv epitopes and were plurifunctional

Broad specificity of T-cell recognition is important even in the context of highly conserved protein regions, because even conserved regions within group M HIV-1 isolates show a low degree of variability and escape.^39,60^ Volunteers receiving the AM, DDDAM, DeDeDeAM and placebo regimens had a median response to 6, 6, 6, and 1/6 peptide pools, respectively, spanning the entire HIVconsv immunogen (Table 2). Note that threefold to fourfold lower frequencies are typically detected in the IFN-γ ELISPOT assay when using cryopreserved relative to fresh PBMC samples (Supplementary Figure S3). In trial HIV-CORE 002, volunteers responded on average to 10 different peptides, of which an average of two (20%) spanned regional junctions and therefore contained epitopes not present in HIV-1 (ref. 28). To estimate the proportion of vaccine-elicited responses across regional junctions in African adults in the current HIV-CORE 004 trial (note that Ad35-GRIN does not stimulate HIVconsv junctional responses), the stimulating 15-mer peptides within pools were identified in a matrix-designed IFN-γ ELISPOT assay. Out of the 50 mapped and confirmed stimulatory 15-mer peptides, three (6%) contained regional junctions (Supplementary Table S2). Using a junction-derived peptide pool JXN and cryopreserved PBMCs in an IFN-γ ELISPOT assay, ~ 12% of the total number of vaccine-induced T cells recognized junctional peptides (Supplementary Figure S4). Overall, the tested vaccine regimens induced broadly-specific T-cell responses recognizing multiple conserved epitopes.

**Figure 5:**
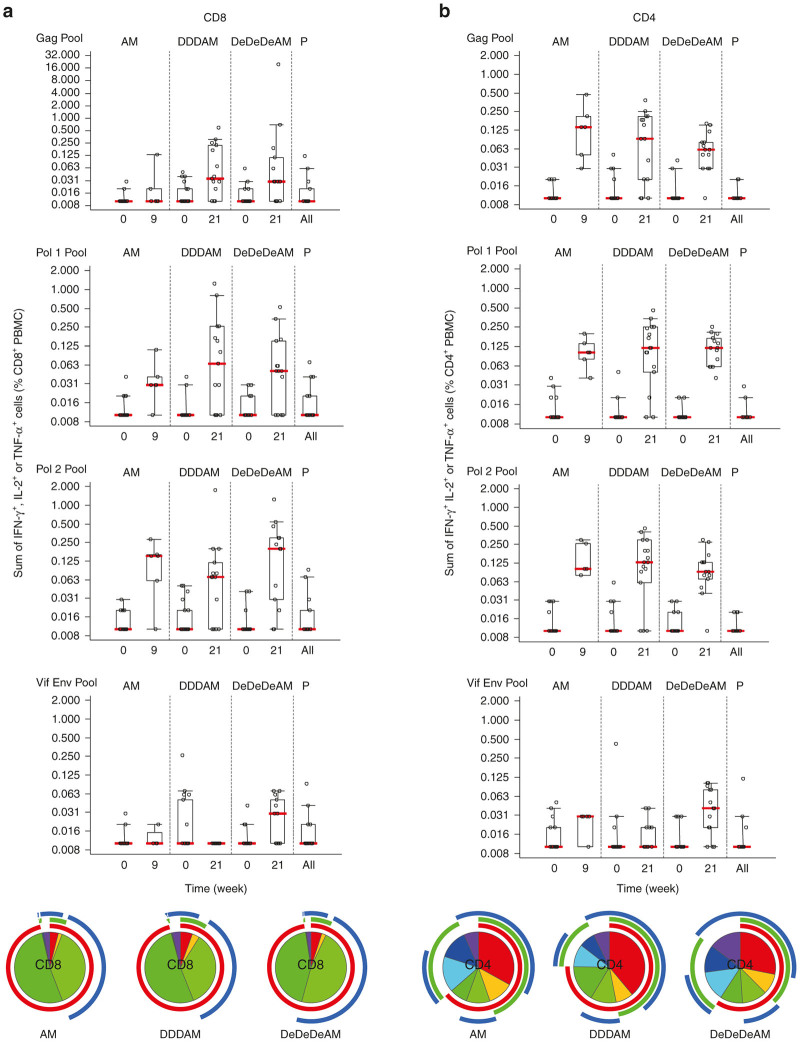
Breadth and plurifunctionality of vaccine-induced CD8^+^ and CD4^+^ T cells. Freshly isolated *ex vivo* peripheral blood mononuclear cells (PBMCs) were assessed for their plurifunctionality (interferon (IFN)-γ, tumor necrosis factor (TNF)-α and interleukin (IL)-2 production) in an intracellular cytokine staining assay using 199 15/11 peptides spanning the entire HIVconsv immunogen, but not region junctions, which were assembled into four pools of Gag, Pol1, Pol2, and Vif+Env. Graphs show the total net (background-subtracted) frequencies of CD8^+^ (**a**) and CD4^+^ (**b**) T cells responding to each cytokine at prevaccination and 1 week after the modified vaccinia virus Ankara (MVA).HIVconsv administration. Pie charts below show for each regimen separately the number of different cytokines that CD8^+^ (**a**) and CD4^+^ (**b**) T cells produced in response to all four peptide pools and include all positive and negative samples. Colors indicate the proportions of HIVconsv-specific cells producing 1 (purple, navy blue, and turquois), 2 (cyan, green, and yellow) and 3 (red) cytokines for the pie slices and IFN-γ (red), IL-2 (green), and TNF-α (blue) for the pie chart arcs.

Production of IFN-γ, TNF-α, and IL-2 upon peptide-pool restimulation of freshly collected and isolated PBMCs was examined by polychromatic flow cytometry at the KAVI-ICR laboratory. HIVconsv-derived peptides were combined into pools Gag, Pol1, Pol2, and Vif+Env and induction of broadly specific CD8^+^ and CD4^+^ T-cell responses within the HIVconsv immunogen was confirmed with a fraction of cells in each population capable of producing all three measured cytokines (Figure 5 and Supplementary Figure S5).

### Vaccine-elicited CD8^+^ effector cells inhibited *in vitro* several viruses of clades A, B, C, and D

**Figure 6:**
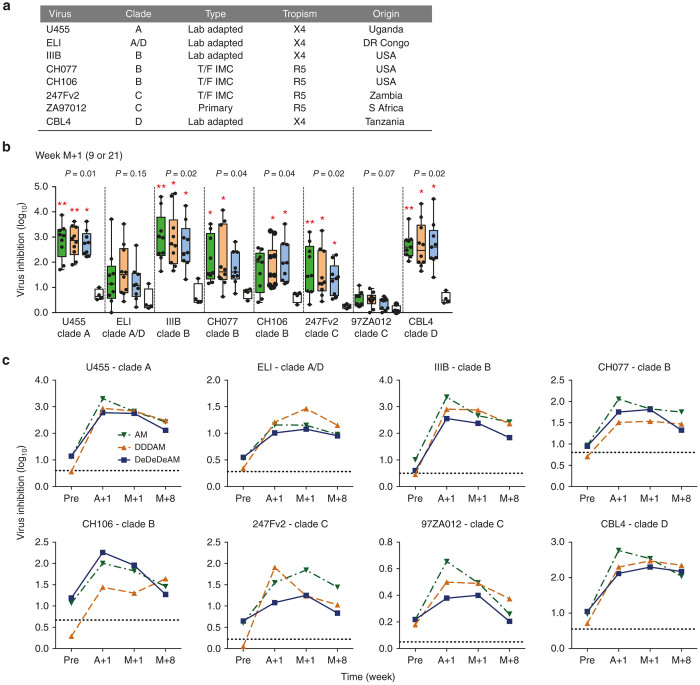
Broad inhibition of HIV-1 replication *in vitro* in viral inhibition assay (VIA). (**a**) A panel of eight viruses used to assess the inhibitory capacity of vaccine-induced CD8^+^ T-cell effectors. (**b**) The M+1 time point was used to compare the inhibitory capacity for the three regimens AM (week 9), DDDAM, and DeDeDeAM (week 21) and placebo. The data are presented as a line for median with 5–95th percentile box showing all data. Green – AM; Orange – DDDAM; Blue – DeDeDeAM; and White - placebo. The Kruskal–Wallis test (analysis of variance (ANOVA)) was used for each virus to determine the approximate *P*-values shown above the graph followed by multiple comparisons of vaccine groups against the placebo group corrected by Dunn’s test (*P < 0.05; **P < 0.01). (**c**) Comparison of the kinetics of the inhibitory activity induction between regimens is shown individually for all eight viruses. Data are presented as group medians for each time point. Green ∇ – AM; Orange Δ – DDDAM; Blue ■ – DeDeDeAM; dotted line shows average placebo inhibition over all time points.

CD8^+^ T-cell effectors induced by the three regimens were tested for inhibition of replication of eight HIV-1 variants: lab adapted U455, ELI, IIIB, and CBL4; transmitted/founder viruses CH077, CH106, and 247Fv2; and primary isolate ZA97012 (Figure 6a). Ten vaccine and two placebo recipients were randomly selected from each Group/regimen. Their frozen samples from prevaccination, post Ad35-GRIN, 1 and 8 weeks after MVA.HIVconsv were used to generate CD8^+^ T-cell effectors and CD4^+^ autologous targets. Group median peak inhibition after the full vaccination regimens showed a statistically significant growth inhibition relative to the placebo controls for six out of eight viruses across all tested clades A, B, C, and D; for the two hardest-to-inhibit viruses ELI and 97ZA012, there was a clear inhibitory trend (Figure 6b,c). Vaccine recipients inhibited an average of 4.1, 4.6, and 3.1 of eight viruses for the AM, DDDAM and DeDeDeAM regimens, respectively. Interestingly, the kinetics of the development of inhibitory responses suggested that for many volunteers, close-to-maximum anti-HIV-1 activity was reached already after the Ad35-GRIN administration (Figure 6,c). For no virus was the post-Ad35-GRIN inhibition significantly different from that detected after the subsequent MVA.HIVconsv boost (Wilcoxon rank sum test) (Figure 6c). Thus, all three AM, DDDAM and DeDeDeAM vaccine regimens administered to healthy African adults induced CD8^+^ T-cell effectors capable of inhibiting the growth of HIV-1’s from four major global clades.

## Discussion

For prophylactic vaccines, pre-expanding the CD8^+^ T-cell effector memory to conserved protein regions matching most circulating HIV-1 variants may enable a first wave of the T-cell response stimulated by new virus exposure to slow or stop the incoming virus effectively,^2^ thus providing the host a critical advantage over the transmitted/founder viruses. Here, we report on the safety and immunogenicity of conserved-region vaccines, plasmid pSG2.HIVconsv DNA without (D) or with (De) electroporation and poxvirus MVA.HIVconsv (M),^21,28^ combined with human adenovirus Ad35-GRIN (A)^55,56^ for administration to healthy African adults. This phase 1/2a trial, HIV-CORE 004, demonstrated that all the regimens AM, DDDAM, and DeDeDeAM were well tolerated and induced high frequencies of HIVconsv-specific T cells, which were of broad specificity and capable of *in vitro* proliferation and inhibition of HIV-1 variants from clades A, B, C, and D. The longevity of vaccine-elicited responses will be further analyzed in a followed-up protocol. These results represent a critical intermediate step on the way from testing a vaccine in the North to evaluating the strategy’s acceptability and efficacy in the South, where an effective HIV-1 vaccine is most urgently needed.

All vaccine regimens of the HIV-CORE 004 trial including repeated electroporation of DNA were generally well tolerated. These observations concur well with accumulating clinical data from trials of tens of thousands of volunteers with similar experimental vaccines for HIV-1, malaria, TB, flu, hepatitis, Ebola, and several other diseases.^55,56,61–69^ Therefore, the safety profile to date of the vaccine modalities employed in trial HIV-CORE 004 encourage further development of these vaccines for both prevention and therapy of HIV-1/AIDS.

At the HIV-CORE 004 trial site in Nairobi, IFN-γ ELISPOT and intracellular cytokine staining (ICS) assays were carried out on freshly isolated PBMCs throughout the clinical protocol. The vaccine recipients responded with frequencies of HIVconsv-specific T cells peaking at medians between 2,158 and 3,590 SFU/10^6^ PBMCs (Figure 4b), recognized a median of 6 out of 6 peptide pools for all three regimens (Table 2) and their CD8^+^ and CD4^+^ T cells were plurifunctional (Figure 5); these are all desirable attributes of candidate vaccines for progressing toward efficacy evaluations.^3^ The HIV-CORE 004 T-cell frequencies were lower compared with the HIV-CORE 002 trial in Oxford,^28^ which might be a consequence of the incomplete match between the HIVconsv immunogen delivered by DNA and MVA, and the GRIN immunogen delivered by human adenovirus 35 used in HIV-CORE 004. Lower responsiveness to vaccination in the South may also reflect differences in HLA/other genetics, nutritional status, and previous/concurrent exposure to pathogens different to the North. The HIV-CORE 004 responses compare favorably to those induced by other T-cell vaccines^2^ including adenovirus-vectored MRKAd5 (ref. 70), electroporated multigenic HIVMAG DNA +/− human IL-12-expressing pDNA boosted with Ad35-GRIN/Env vaccine,^55^ and others.^61,65,71–73^ These data reaffirm in the context of African HLA haplotypes and settings, that conserved subdominant epitopes,^47,49–51^ taken out of the context of full-length proteins and delivered by potent heterologous regimens, induce robust T-cell responses. This is an important observation for further advancement of this vaccine strategy.

Following i.m. delivery of the pSG2.HIVconsv DNA, electroporation using the Ichor Medical Systems’ TriGrid device enhanced DNA-induced responses. This initial advantage in the magnitude of DNA priming was not carried over subsequent viral vaccine boosts. The timing of the MVA.HIVconsv boost may have been suboptimal for the electroporation regimen compared with nonelectroporated DNA (Figure 4a) and perhaps a longer interval between the Ad35-GRIN and MVA.HIVconsv vaccines might have resulted in a much larger overall expansion of the HIVconsv-specific T cells. By the end of the clinical protocol at week 44, the frequencies of circulating vaccine-elicited IFN-γ-producing T cells were indistinguishable between the two regimens and similar to the AM regimen. This does not rule out qualitative T-cell differences among individual regimens, e.g. as a consequence of low-dose T-cell priming by DNA, which are a subject of further investigation, and an extended follow-up of HIV-CORE 004 volunteers is ongoing. Thus, these data confirm previous observations of a very modest enhancement of DNA alone vaccination in humans by electroporation, but as yet no obvious overall benefit.^55,74–77^

VIAs, in the absence of a simple T-cell functional correlate of protection, may be the best *in vitro* predictor of *in vivo* CD8^+^ T-cell protective capacity against HIV-1.^36^ This is supported by correlation of VIA activities with control of HIV-1 load in long-term nonprogressors,^9,32,33,78^ prediction of the rate of CD4^+^ cell decline in HIV-1 infection^35^ and limited breadth of inhibition induced by vaccines MRKAd5, VRC DNA/Ad5, and ALVAC/AIDSVAX.^30^ However, correlation with a real vaccine-induced benefit in humans remains to be demonstrated. These conclusions are backed by our demonstration that antigen-nonspecific antibody expansion of CD8^+^ effector cells does not skew the T-cell composition and is, therefore, a valid approach for assessing CD8^+^ T cell-mediated HIV-1 inhibition.^27,28,30^ Here, one half of vaccine and placebo recipients, randomly selected from each group, were tested against a panel of eight HIV-1 isolates selected across the four major clades of group M. Relative to placebo, the vaccine recipients showed an overall broad inhibition of six viruses and a trend of inhibition for the other two most difficult-to-inhibit ones. Two interesting points were noted. First, not all viruses were inhibited with the same efficiency (Figure 6b) suggesting that, similar to classification of viruses according to the antibody neutralization sensitivity into tiers 1–3, perhaps analogous classification may apply for T-cell inhibition. This is similar to HIV-CORE 002, where we showed that the level of inhibition was independent of the ability of the viruses to replicate in autologous CD4^+^ cells.^28^ Second, for all viruses and regimens, a level of inhibition statistically inseparable from that after the last MVA.HIVconsv boost was achieved by the Ad35-GRIN (Figure 6a); this was an unexpected observation as previously VIA inhibition correlated well with IFN-γ ELISPOT peak frequencies.^27,28^ Future iterative improvements of T-cell vaccines will focus on enhancing the VIA inhibition. Indeed, the first generation HIVconsv conserved regions, which are constructed as alternating clade consensus sequences, were conceived using the HIV Sequence Database over a decade ago.^21^ The second generation tHIVconsvX vaccines focus immune responses on computer-redefined conserved regions of HIV-1 Gag and Pol only (leaving Env out completely because of its variability and absence of protective epitopes) with efficacy further enhanced by an improved match to global circulating viruses through using a bivalent mosaic,^37,60^ by incorporating conserved and protective epitopes identified in treatment-naive patient cohorts on four continents^22,23^ and by minimizing induction of responses against irrelevant junctional epitopes.^39^ The tHIVconsvX vaccines are now in the pipeline for clinical testing.

In conclusion, HIV-CORE 004 is a critical trial for evaluating the conserved-region T-cell vaccine strategy. Confirmation of the vaccine tolerability and acceptability by the African volunteers, and demonstration of broad inhibition of HIV-1 variants by vaccine-elicited T cells in a low-medium income country are encouraging and are an important stepping stone toward a timely evaluation of the improved second generation conserved mosaic vaccines in efficacy studies, whereby the T-cell vaccines will be likely accompanied by an Env vaccine component. Finally, conducting the trial and trial associated activities and assays in Nairobi contributed to the preparedness of the KAVI-ICR and Kangemi clinic for future larger studies.

## Materials and Methods

### Ethical and regulatory approvals

The HIV-CORE 004 trial was sponsored by University of Oxford. Approvals for HIV-CORE 004 were granted by the Oxford Tropical Research Ethics Committee (OxTREC), University of Oxford, UK (ref. no.: 1006–13), Stockholm Regional Ethics Committee, Stockholm, Sweden (ref. no.: 2009/1591-31/1) and by Kenyatta National Hospital/University of Nairobi Ethics and Research Committee (KNH/UoN-ERC), Nairobi, Kenya (ref. no.: P11/01/2013) and by the Kenya national regulatory authority, the Pharmacy & Poisons Board (PPB), Kenya (ref. no.: PPB/ECCT/13/07/01/2013). The study was conducted according to the principles of the Declaration of Helsinki (2008) and complied with the International Conference on Harmonization Good Clinical Practice guidelines. The trial is registered on the Pan African Clinical Trials Registry (ref. no.: PACTR201403000794397).

### Study design and subjects

Trial HIV-CORE 004 was a double-blind, randomized, placebo-controlled phase 1/2a study. The trial was conducted at the KAVI-Institute for Clinical Studies (KAVI-ICR), Kangemi site, Nairobi, Kenya between April 2014 and August 2015. Healthy HIV-1/2-negative males and nonpregnant females aged 18–50 years were invited to participate in the study. All volunteers were at low risk of HIV-1 infection and all gave written informed consent before participation. There was no selection of volunteers on the basis of pre-existing neutralizing antibodies to human adenovirus (HAdV)-35 or HAdV-5 before enrolment.

### Safety monitoring

An independent Data Monitoring and Ethics Committee chaired by Philip Bejon (University of Oxford, Kilifi) and including Eduard Sanders (University of Oxford, Kilifi) and Paul Klenerman (University of Oxford, Oxford) oversaw the study safety data. CLINWIN (www.clinwinresearch.com) and IAVI regularly monitored the study records. Pause criteria were predetermined in the Study Protocol.

### Randomization 

The randomization schedule was prepared by the Data Coordinating Center (DCC) of the EMMES Corporation (www.emmes.com). Volunteers were randomly assigned to one of three groups described in Figure 1b and randomized to vaccine or placebo in a 5:1 ratio, using a block size of five. Study site staff (except the pharmacist), volunteers, laboratory staff, and medical monitors were blinded to assignment to vaccine or placebo. Investigators at the study sites enrolled volunteers via an electronic system (administered by the DCC), where allocation codes were assigned consecutively to eligible volunteers at the time of first vaccination.

### Vaccines

The pSG2.HIVconsv DNA vaccine was produced by the Clinical Biotechnology Centre, Bristol Institute for Transfusion Science, University of Bristol, UK and formulated in phosphate buffered saline pH 7.4 at 4.0 mg/ml. The Ad35-GRIN was produced by Transgene SA, Illkirch, France and diluted in formulation TG0004 S01 Buffer to 2 × 10^11^ vp/ml. The vaccine and diluent were stored under GMP at and packaged specifically for the HIV-CORE 004 study with a compliant label by B&C (Brussels, Belgium). The MVA.HIVconsv vaccine was produced by IDT Biologika GmbH, Dessau-Rosslau, Germany and diluted in a formulation buffer to 5.5 × 10^8^ PFU/ml. Placebo was sterile normal saline provided by pharmacy. All vaccines were stored at or below −70°C until use.

### Vaccinations

Vaccines were thawed not more than 1 hour prior to injection and kept on ice. All vaccines were administered into the deltoid muscle of both arms by an i.m. needle injection at the following doses: 4 mg of pSG2.HIVconsv DNA, which was administered with or without electroporation using of the TriGrid Delivery System of Ichor Medical Systems, San Diego, CA, USA, 5 × 10^10^ vp of Ad35-GRIN and 2 × 10^8^ PFU MVA.HIVconsv. The regimens are schematically depicted in Figure 1b.

### Peptides and antigens

HIVconsv-derived 15-mer peptides overlapping by 11 aa (Ana Spec, San Jose, CA 95131) were reconstituted to 40 mg/ml in dimethylsulfoxide (DMSO) and diluted to working stock solutions of 4 mg/ml in phosphate-buffered saline. For ELISPOT assays, peptides were combined into six pools, P1–P6, of 32–36 peptides per pool in R10 (RPMI 1460 supplemented with 10% FBS, 2 mmol/l L-glutamine, 1 mmol/l sodium pyruvate, 10 mmol/l *N*-2-hydroxyethylpiperazine-*N9-2*-ethanesufonic acid (HEPES) and penicillin-streptomycin antibiotics; Sigma Aldrich, St. Louis, MO) and 2×-concentrated stocks of 3 µg/ml were prepared and aliquoted into peptide plates along with the positive and negative controls. For frozen ELISPOT assay, peptides were arranged into seven pools comprising nonjunctional peptides in P1–P6 and junctional peptides in JXN. For identification of stimulatory 15-mers, peptides were arranged into 3-D matrix pools. For ICS studies, the peptides were combined into four pools covering Gag, Pol (two pools), and Env+Vif. A pool of FEC (“Flu”, EBV and CMV) peptides consisting of 32 previously defined CD8^+^ T-cell epitopes from influenza virus, Epstein-Barr virus, and cytomegalovirus (CEF; NIH AIDS Research and Reference Program) was reconstituted in dimethylsulfoxide and used at a final concentration of 1 µg/ml as a positive control. All peptide stocks and plates were stored at −80^o^C until use.

### Isolation and cryopreservation of PBMCs

Blood was drawn into heparinized vacutainers (Becton Dickinson, San Jose, CA) and processed by the laboratory within 6 hours. Standard procedures were used for cryopreservation.^55,56^

### *Ex vivo* IFN-γ ELISPOT assay

Freshly isolated PBMCs were used. ELISPOT plates (S5EJ044I10; Merck Millipore, Darmstadt, Germany) prewetted for 1 minute with 15 µl of 35% ethanol were coated overnight at 4^o^C with anti-IFN-γ antibody (10 µg/ml in phosphate-buffered saline; clone 1-D1K; Mabtech, Nacka Strand, Sweden). Prior to use, plates were washed with phosphate-buffered saline and blocked with R10 for a minimum of 1 hour at 37^o^C. The PBMC were plated out at 2 × 10^5^ cells/well in 50 µl for the majority of time points and at 1 × 10^5^ cells/well at peak time points. For HIVconsv, pools P1–P6 responses were detected in triplicate wells. Six negative no-peptide control wells were cells cultured in R10 supplemented with 0.45% dimethylsulfoxide Positive controls in triplicate wells were cells cultured with 10 µg/ml phytoheamagglutinin (PHA) (Sigma Aldrich, Dorset, UK) or a pool of FEC (flu/EBV/CMV) peptides at 1 µg/ml. The cells were incubated overnight at 37°C in 5% CO_2_. Spots were visualized using biotin anti-IFN-γ combined with streptavidin/alkaline phosphate (both from Mabtech) and the color was developed using substrate BCIP/NBT^Plus^ (Mabtech). The reaction was stopped after 5 minutes by washing under the tap. The plates were air dried overnight and the spots counted using an AID ELISpot Reader and version 5.0 software (AID GmbH). After quality control of the data (see Statistical Analysis below), the mean number of SFU in no-peptide wells were subtracted from test wells and the results were expressed as the median net SFU/10^6^ PBMC. Responses at least 38 SFU/10^6^ PBMCs above background and at least 4× background were score as positive. Electronic records for each plate were retained.

### Intracellular cytokine staining assay

Flow cytometry was performed as described previously.^28,55^ Antigen-specific phenotypes and cytokine secretion profiles were assessed using a qualified polychromatic flow cytometry panel. Freshly isolated PBMCs were coincubated with peptide pools Gag, Pol1, Pol2, and Vif+Env matched to HIVconsv, 1 μg/ml SEB (Sigma-Aldrich) or mock stimuli, BD Golgistop (Becton Dickinson) and Brefeldin A (Sigma-Aldrich, Dorset, UK) for 6 hours at 37°C. Cells were stained for viability with LIVE/DEAD Fixable Violet Dead Cell Stain Kit (Invitrogen, Eugene, OR), and then surface stained by anti-CD4 PeCF594, anti-CD8 BV421, anti-CD3 pacific blue (Invitrogen, Paisley, UK). Finally, intracellular staining was performed with anti-CD3 APC-H7 (Invitrogen, Paisley, UK), anti-IFN-γ APC, anti-TNF-α fluorescein isothiocyanate(FITC) and anti-IL-2 PE (Becton Dickinson) washed and acquired on the same day. At least 750,000 events per sample were acquired on a custom-built BD LSR II cytometer. Data were analyzed and presented using FlowJo (version 9.9 Treestar, Ashland, OR) and plurifunctionality was determined using SPICE (Bioinformatics and Computational Biosciences Branch, NIAID, NIH, Bethesda, MD).

### Viral inhibition assay

A VIA assay was qualified for use in vaccine trials as described previously.^27,28,30,34^ Cryopreserved and thawed PBMCs were resuspended at a density of 1 × 10^6^ cells/ml in R10 medium supplemented with 50 U of IL-2 and 0.5 μg/ml CD3/CD4 or CD3/CD8 bispecific antibodies (provided by Johnson Wong, Harvard Medical School) for generation of CD8^+^ or CD4^+^ T cells, respectively, for 7 days. Culture volumes were doubled at days 3 and 6 by addition of fresh medium and IL-2. CD4^+^ T cells were infected at a multiplicity of infection of 0.01 for 4 hours with a panel of 8 HIV-1 isolates-IIIB (accession number K03455, subtype B), ELI (K03454, subtype A/D), U455 (M62320, subtype A), and 97ZA012 (AF286227, subtype C) provided by the NIH AIDS reagent repository, CH077 (JN944909, subtype B), CH106 (JN944897, subtype B), 247FV2 (NA, subtype C) provided by George Shaw, University of Birmingham, AL, USA and CBL4 (NA, subtype D) kindly provided by the National Institutes of Biological Standards and Control, UK. Gag p24 in the supernatant was measured on day 13 by enzyme-linked immunosorbent assay (ELISA) (PerkinElmer, Waltham, MA). CD8^+^ T cell-mediated inhibition was expressed as the log_10_ reduction in p24 content of day 13 CD8^+^ and CD4^+^ T-cell cocultures, compared with infected CD4^+^ T cells alone. For clinical trial volunteers, antibody-expanded prevaccination CD4^+^ T cells were used as common targets for HIV-1 infection in cocultures with pre- and post-vaccination CD8^+^ T cells.

### Statistical analysis

Once the sample size of 72 (60 vaccine and 12 placebo) across three groups was determined, the power calculations and detectable effect sizes were based on the sample size. Prior to immunogenicity analysis, ELISPOT assay responses were screened for extreme outliers and any individual well with a value inconsistent with the median of the replicates was removed before averaging. Following the exclusion of outliers, samples were excluded if the average response in mock wells was ≥ 50 SFU/10^6^ PBMC or the average PHA was < 400 SFU/10^6^ PBMC. Assays with high or low FEC responses were also failed by the laboratory. Responses were assumed to be non-Gaussian in distribution, thus Wilcoxon rank sum tests were used for comparisons throughout and medians (range) are shown or boxplots presented. We analyzed the maximum/peak of the total response *i.e.*, the summed responses to pools 1–6), and, as a measure of overall response, we used the AUC statistically compared using Wilcoxon rank sum test. Data management and analysis was conducted in Stata (version 13.1) (StataCorp PL; College Station, Texas, USA) and R (version 3.2.3) (The R Foundation for Statistical Computing; Vienna, Austria). The safety comparisons were based on the maximum severity per volunteer and analyses were performed using SAS version 9.2 (SAS, Cary, NC). For all analyses, two-tailed *P*-values were used and *P*-value < 0.05 was considered statistically significant unless corrected for multiple comparisons.

## Figures and Tables

**Table 1:** Trial HIV-CORE 004 demographics

	*AM*	*DDDAM*	*DeDeDeAM*	*Placebo*	*Total*
Number of volunteers	20	20	20	12	72
Gender					
Female	14 (70.0%)	8 (40.0%)	10 (50.0%)	9 (75.0%)	41 (56.9%)
Male	6 (30.0%)	12 (60.0%)	10 (50.0%)	3 (25.0%)	31 (43.1%)
Race					
Black Africans	20 (100.0%)	20 (100.0%)	20 (100.0%)	12 (100.0%)	72 (100.0%)
Ethnicity					
Non-Hispanic and Non-Latino	20 (100.0%)	20 (100.0%)	20 (100.0%)	12 (100.0%)	72 (100.0%)
Age (years)					
Missing information	0	0	0	0	0 (0.0%)
Mean	27.4	26.5	31.1	29.3	28.5
Range	20–39	19–44	18–49	21–41	18–49
Height (cm)					
Missing information	1 (5.0%)	0	0	0	1 (1.4%)
Mean	165	168	166	163	166
Range	155–185	153–181	153–178	147–183	147–185
Body Mass (kg)					
Missing information	1 (5.0%)	0	0	0	1 (1.4%)
Mean	68	65	66	77	68
Range	47–96	50–109	50–87	58–111	47–111

**Table 2:** Peak magnitudes and breadth of vaccine-elicited T-cell responses against HIVconsv-derived peptide pools

	*P1*	*P2*	*P3*	*P4*	*P5*	*P6*	*Number of recognized * *pools per participant*
Median (range)							
AM	399 (143–9,970)	102 (18–750)	478 (19–1,193)	121 (28–877)	405 (11–3,207)	386 (41–2,727)	6 (2–6)
DDDAM	1,035 (113–2,809)	338 (26–2,307)	643 (92–4,519)	329 (38–808)	477 (53–2,190)	728 (82–2,153)	6 (5–6)
DeDeDeAM	491 (64–5,073)	315 (62–2,477)	457 (141–16,080)	203 (84-11,213)	373 (111–1,616)	459 (39–2,863)	6 (6–6)
Placebo	25 (13–86)	23 (3–88)	26 (13–59)	20 (7–88)	21 (3–143)	26 (3–134)	1 (0–4)
