# Supplementary material for: Broad HIV-1 inhibition in vitro by vaccine-elicited CD8+ T cells in African adults
Source: Mol Ther Methods Clin Dev. 2016 Aug 31;3:16061–. doi: 10.1038/mtm.2016.61 (PMC5006719; doi:10.1038/mtm.2016.61)
Supplement: Supplementary Figures and Tables [file mtm201661-s1.pdf]

Supplementary Table S1 HLA class I types of trial volunteers

	Volunteer No.	HLA-A		HLA-B		HLA-C	
Group 1	1767	02:01:01	68:02:01	07:02:01	53:01:01	04:01:01	15:05:01
	1772	02:02	32:01:01	15:67	58:02	06:02:01	14:02:03
	1773	02:01:01	36:01	15:03:01	53:01:01	02:10	04:01:01
	1777	01:01:01	23:01:01	07:02:01	57:01:01	07:01:01	07:02:01
	1781	30:02:01	74:01:01	53:01:01	58:02	06:02:01	
	1782	03:01:01	30:02:01	15:10:01	35:01:01	03:04:02	04:01:01
	1789	02:05:01	74:01:01	58:01:01	58:02	06:02:01	
	1792	30:01:01	74:01:01	42:01:01	58:02	06:02:01	17:01:01
	1798	02:01:01	23:01:01	53:01:01	57:03:01	04:01:01	08:02:01
	1822	01:01:01	68:02:01	37:01:01	45:01:01	06:02:01	
	1835	23:01:01	29:02:01	07:02:01	45:01:01	06:02:01	07:02:01
	1837	30:01:01		15:10:01	53:01:01	03:04:02	04:01:01
	1843	01:01:01	68:02:01	13:02:01	58:01:01	03:02:01	18:01:01
	1848	02:14	34:02:01	18:03	44:03:01	04:01:01	
	1850	02:01:01		27:03	39:10:01	02:02:02	12:03:01
	1855	36:01		15:16:01	53:01:01	04:01:01	16:01:01
	1857	02:01:01	23:01:01	42:01:01	53:01:01	04:01:01	17:01:01
	1859	03:01:01	36:01	47:03	53:01:01	04:01:01	07:01:01
	1866	02:01:01	33:03:01	15:03:01	53:01:01	02:10	04:01:01
	1867	66:01:01	68:02:01	15:03:01	47:03	02:10	07:01:01
	1765	66:02	68:02:01	15:10:01	58:01:01	03:04:02	07:01:01
	1828	23:01:01		15:03:01		02:10	12:03:01
	1833	30:02:01	74:01:01	08:01:01	58:02	03:04:01	06:02:01
	1864	29:02:01	68:02:01	07:02:01	42:01:01	07:02:01	17:01:01
Group 2	1762	02:01:01	68:01:01	15:03:01	15:17:01	02:10	17:01:01
	1768	29:02:01	34:02:01	39:10:01	40:12	04:04:01	12:03:01
	1770	01:01:01	68:02:01	47:03	81:01:01	07:01:01	18:01:01
	1774	24:02:01	30:01:01	18:01:01	51:01:01	07:01:01	16:01:01
	1775	02:05:01	30:09	15:10:01	81:01:01	03:04:02	04:01:01
	1783	01:01:01	74:01:01	15:03:01	37:01:01	02:10	06:02:01
	1786	29:02:01	31:01:02	18:03	27:03	02:02:02	04:01:01
	1788	02:05:01	23:01:01	27:03	49:01:01	02:02:02	07:01:01
	1797	02:05:01	30:01:01	42:01:01	51:01:01	16:01:01	17:01:01
	1829	02:01:01	30:01:01	42:01:01	51:01:01	16:01:01	17:01:01
	1832	26:01:01	34:02:01	07:05:01	53:01:01	04:01:01	08:02:01
	1834	03:01:01	26:01:01	15:03:01	57:03:01	04:01:01	07:01:01
	1838	02:05:01	68:02:01	15:03:01	57:03:01	02:10	07:01:01
	1841	68:02:01	74:01:01	13:02:01	35:01:01	04:01:01	06:02:01
	1851	01:01:01	01:03	44:03:02	56:01:01	01:02:01	07:01:01
	1853	29:01:01	66:01:01	42:01:01	58:02	06:02:01	17:01:01
	1858	01:02	29:02:01	39:10:01	58:01:01	03:02:01	12:03:01
	1863	68:01:01	68:02:01	14:02:01	15:10:01	03:04:02	08:02:01
	1868	03:01:01	66:01:01	15:03:01	58:02	06:02:01	
	1869	68:02:01		07:02:01		07:02:01	
	1780	01:03	66:01:01	57:03:01	58:02	06:02:01	07:01:01
	1795	30:02:01	32:01:01	14:02:01	58:01:01	06:02:01	08:02:01
	1846	01:01:01	02:01:01	18:01:01	37:01:01	06:02:01	07:04:01
	1862	02:01:01	02:05:01	35:01:01	42:01:01	04:01:01	17:01:01

Supplementary Table S1 Continued.

	Volunteer No.	HLA-A		HLA-B		HLA-C	
Group 3	1764	29:15	30:04:01	45:01:01	58:01:01	06:02:01	16:01:01
	1766	02:01:01	03:01:01	15:03:01	42:01:01	02:10	17:01:01
	1769	02:01:01	68:02:01	07:02:01	15:03:01	02:10	07:02:01
	1771	02:14	23:01:01	14:02:01	18:03	04:01:01	08:02:01
	1778	66:01:01	74:01:01	45:01:01	58:02	06:02:01	
	1784	03:01:01	74:01:01	08:01:01	51:01:01	04:01:01	07:01:01
	1790	02:05:01	30:02:01	14:02:01	58:01:01	06:02:01	08:02:01
	1791	02:01:01	68:02:01	07:02:01	27:03	02:02:02	07:02:01
	1823	02:05:01	36:01	27:03	53:01:01	02:02:02	04:01:01
	1824	01:03	02:02	58:01:01	58:02	03:02:01	06:02:01
	1831	01:09	29:02:01	15:17:01	44:15	04:07	17:01:01
	1836	23:01:01	33:01:01	15:10:01	45:01:01	03:04:02	06:02:01
	1840	26:01:01	68:02:01	53:01:01	81:01:01	04:01:01	
	1842	02:01:01	29:02:01	42:01:01	53:01:01	06:02:01	17:01:01
	1849	01:01:01	02:01:01	15:03:01	27:26	02:02:02	02:10
	1852	23:01:01	66:01:01	08:01:01	44:03:01	04:01:01	07:01:01
	1860	02:02	29:02:01	42:01:01	57:03:01	07:01:01	17:01:01
	1865	01:01:01	68:02:01	13:02:01	47:01:01	06:02:01	
	1870	01:01:01	03:01:01	41:01	58:02	06:02:01	07:01:01
	1871	02:14	68:02:01	07:02:01	15:03:01	07:01:01	07:02:01
	1776	30:02:01	36:01	15:03:01	15:16:01	02:10	16:01:01
	1821	30:04:01	68:02:01	45:01:01	47:03	07:01:01	08:04:01
	1847	29:02:01	32:01:01	42:01:01	58:02	04:01:01	17:01:01
	1861	01:01:01		45:01:01	49:01:01	06:02:01	

Supplementary Table S2 Identified and confirmed stimulatory 15-mer peptides derived from HIVconsv using frozen unexpanded PBMC samples[a]

Gr	ID	Week	Peptide No.	Protein of Origin/Clade	Sequence	Magnitude (SFU/10^6 PBMC)
1	1767	10	HIVCON 182	ENV/D	LGAAGSTMGAASMTL	367
1	1777	10	HIVCON 88	POL/C	GSPAIFQSSMTKILE	186
1	1781	10	HIVCON 163	POL/B	KTAVQMAVFIHNFKR	57
1	1781	10	HIVCON 164	POL/B	VQMAVFIHNFKRKGGI	77
1	1789	10	HIVCON 160	POL/B	IIGQVRDQAEHLKTA	194
1	1789	10	HIVCON 164	POL/B	VQMAVFIHNFKRKGGI	101
1	1789	10	HIVCON 165	POL/B	FIHNFKRKGGIGGYS	51
1	1850	10	HIVCON 15	GAG/D	IYKRWIILGLNKIVR	1980
1	1864	10	HIVCON 159	POL/B	ELKKIIGQVRDQAEH	118
1	1864	10	HIVCON 163	POL/B	KTAVQMAVFIHNFKR	58
1	1864	10	HIVCON 164	POL/B	VQMAVFIHNFKRKGGI	72
1	1864	10	HIVCON 165	POL/B	FIHNFKRKGGIGGYS	782
2	1762	22	HIVCON 113	POL/A-B	SKDLIAEIQ-YWQATW[b]	178
2	1774	22	HIVCON 82	POL/C	TIPSINNETPGIRYQ	73
2	1774	22	HIVCON 83	POL/C	INNETPGIRYQYNVL	143
2	1788	22	HIVCON 15	GAG/D	IYKRWIILGLNKIVR	190
2	1786	22	HIVCON 15	GAG/D	IYKRWIILGLNKIVR	536
2	1832	22	HIVCON 87	POL/C	KQGWKGSPAIFQSSMT	550
2	1832	22	HIVCON 88	POL/C	GSPAIFQSSMTKILE	585
2	1834	22	HIVCON 1	GAG/C	MEEKAFSPEVIPMFT	250
2	1834	22	HIVCON 77	POL/C	VGDAYFSVPLDEGFR	463
2	1834	22	HIVCON 78	POL/C	YFSVPLDEGFRKYTA	940
2	1834	22	HIVCON 79	POL/C	PLDEGFRKYTAFTIP	53
2	1841	22	HIVCON 55	POL/B	GPENPYNTPVFAIKK	411
2	1841	22	HIVCON 78	POL/C	YFSVPLDEGFRKYTA	1274
2	1841	22	HIVCON 79	POL/C	PLDEGFRKYTAFTIP	371
2	1841	22	HIVCON 163	POL/B	KTAVQMAVFIHNFKR	291
2	1841	22	HIVCON 164	POL/B	VQMAVFIHNFKRKGGI	258
2	1841	22	HIVCON 177	POL/C	RKAKIIRDYGKQMAG	98
2	1841	22	HIVCON 178	POL/C	IIRDYGKQMAGADCV	824
2	1863	22	HIVCON 13	GAG/C-D	EEAAEWDR-IYKRWII[b]	80
2	1863	22	HIVCON 62	POL/B	GPENPYNTPVFAIKK	1136
2	1863	22	HIVCON 63	POL/B	PYNTPVFAIKKKDST	933
3	1764	22	HIVCON 12’	GAG/C	DTINEEAAEWDR	340
3	1766	22	HIVCON 176	POL/C	VVPRRKAKIIRDYGK	344
3	1766	22	HIVCON 177	POL/C	RKAKIIRDYGKQMAG	79
3	1771	22	HIVCON 1	GAG/C	MEEKAFSPEVIPMFT	61
3	1771	22	HIVCON 154	POL/A	IPAETGQETAYFLLK	161
3	1771	22	HIVCON 163	POL/B	KTAVQMAVFIHNFKR	128
3	1771	22	HIVCON 164	POL/B	VQMAVFIHNFKRKGGI	106
3	1784	22	HIVCON 88	POL/C	GSPAIFQSSMTKILE	376
3	1784	22	HIVCON 89	POL/C	IFQSSMTKILEPFRA	79
3	1784	22	HIVCON 163	POL/B	KTAVQMAVFIHNFKR	456
3	1784	22	HIVCON 164	POL/B	VQMAVFIHNFKRKGGI	609
3	1790	22	HIVCON 137’	POL/D	KVLFLDGIDKAQ	168
3	1790	22	HIVCON 137	POL/D-A	KVLFLDGIDKAQ-AKE[b]	145
3	1790	22	HIVCON 136	POL/D	SQGIRKVLFLDGIDKA	137
3	1836	22	HIVCON 40	POL/B	ILIEICGHKAIGTVL	223
3	1836	22	HIVCON 41	POL/B	ICGHKAIGTVLVGPT	163
3	1836	22	HIVCON 174	POL/C	IQDNSDIKVVPRRKA	318

a - For this analysis, frozen samples from week 10 for Group 1 and from week 22 for Groups 2 and 3 were used; b - Hyphen indicates a junction between two adjacent regions.

GRIN – Full-length proteins of clade A Gag, RT, Integrase and Nef
HIVconsv - Conserved regions of alternating clade consensus

```
MAARASILSGGKLDAWEKIRLRPGGKKKYRLKHLVWASRELDRFALNPSLLETTEGCQQIMNQLQP
------------------------------------------------------------------

AVKTGTEEIKSLFNTVATLYCVHQRIDVKDTKEALDKIEEIQNKSKQKTQQAAADTGDSSKVSQNY
------------------------------------------------------------------

PIIQNAQGQMIHQNLSPRTLNAWVKVIEEKAFSPEVIPMFSALSEGATPQDLNVMLNIVGGHQAAM
---------------------------EEKAFSPEVIPMFTALSEGATPQDLNTMLNTVGGHQAAM

QMLKDTINEEAAEWDRLHPVQAGPIPPGQIREPRGSDIAGTTSTPQEQLQWMTGNPPIPVGNIYKR
QMLKDTINEEAAEWDR----------------------------------------------IYKR

WIILGLNKIVRMYSPVSILDIKQGPKEPFRDYVDRFFKALRAEQATQDVKGWMTETLLVQNANPDC
WIILGLNKIVRMYSPVSILDIRQGPKEPFRDYVDRF------------------------------

KSILKALGSGATLEEMMTACQGVGGPGHKARVLAEAMSQAQQTNIMMQRGNFRGQKRIKCFNCGKE
------------------------------------------------------------------

GHLARNCRAPRKKGCWKCGKEGHQMKDCTERQANFLGKIWPSSKGRPGNFPQSRPEPTAPPAELFG
---ARNCRAPRKKGCWKCGKEGHQMKDCTERQANFLGKIWPS------------------------

MGEGIASLPKQEQKDREQVPPLVSLKSLFGNDPLSQGS----------------------------
---------------------------------------------RWKPKMIGGIGGFIKVRQYDQ

--------------------------------------PISPIETVPVTLKPGMDGPKVKQWPLTE
ILIEICGHKAIGTVLVGPTPVNIIGRNLLTQIGCTLNFPISPIETVPVKLKPGMDGPKVKQWPLTE

EKIKALTEICTEMEKEGKISKIGPENPYNTPIFAIKKKDSTKWRKLVDFRELNKRTQDFWEVQLGI
EKIKALVEICTEMEKEGKISKIGPENPYNTPVFAIKKKDSTKWRKLVDFRELNKRTQDFWEVQLGI

PHPAGLKKKKSVTVLDVGDAYFSVPLDENFRKYTAFTIPSTNNETPGVRYQYNVLPQGWKGSPAIF
PHPAGLKKKKSVTVLDVGDAYFSVPLDEGFRKYTAFTIPSINNETPGIRYQYNVLPQGWKGSPAIF

QSSMTKILEPFRSKNPEIIIYQYMAALYVGSDLEIGQHRTKIEELRAHLLSWGFTTPDKKHQKEPP
QSSMTKILEPFRAQNPEIVIYQYMDDLYVGSDLEIGQHR---------------------------

FLWMGYELHPDKWTVQPIMLPDKESWTVNDIQKLVGKLNWASQIYAGIKVKQLCRLLRGAKALTDI
------------------------------------------------------------------

VTLTEEAELELAENREILKDPVHGVYYDPSKDLVAEIQKQGQDQWTYQIYQEPFKNLKTGKYARKR
--LTEEAELELAENREILKDPVHGVYYDPSKDLIAEIQ----------------------------

SAHTNDVRQLAEVVQKVAMESIVIWGKTPKFKLPIQKETWETWWMDYWQATWIPEWEFVNTPPLVK
----------------------------------------------YWQATWIPEWEFVNTPPLVK
```

Supplementary Figure S1

```
LWYQLEKDPILGAETFYVDGAANRETKLGKAGYVTDRGRQKVVSLTETTNQKTELHAILLALQDSG
LWYQLEK-----------------------------------------------------------

SEVNIVTDSQYALGIIQAQPDRSESELVNQIIEKLIGKDKIYLSWVPAHKGIGGNEQVDKLVSSGI
--------------------------------------------WVPAHKGIGGNEQVDKLVSQGI

RKVLFLDGIDKAQEDHERYHSNWRTMASDFNLPPIVAKEIVASCDKCQLKGEAMHGQVDCSPGIWQ
RKVLFLDGIDKAQ-----------------------AKEIVASCDKCQLKGEAMHGQVDCSPGIWQ

LACTHLEGKVILVAVHVASGYIEAEVIPAETGQETAYFLLKLAGRWPVKVVHTANGSNFTSAAVKA
LDCTHLEGKVILVAVHVASGYIEAEVIPAETGQETAYFLLKLA-----------------------

ACWWANIQQEFGIPYNPQSQGVVASMNKELKKIIGQVRDQAEHLKTAVQMAVFIHNFKRKGGIGGY
-------------------------MNKELKKIIGQVRDQAEHLKTAVQMAVFIHNFKRKGGIGGY

SAGERIIDIIATDIQTKELQKQITKIQNFRVYYRDSRDPIWKGPAKLLWKGEGAVVIQDNSDIKVV
SAGERI----------------------------------WKGPAKLLWKGEGAVVIQDNSDIKVV

PRRKAKILRDYGKQMAGDDCVAGRQDEDRSMGGKWSKGSIVGWPEIRERMRRAPAAAPGVGAVSQD
PRRKAKIIRDYGKQMAGADCV---------------------------------------------

LDKHGAITSSNINNPSCVWLEAQEEEEVGFPVRPQVPLRPMTYKGAFDLSHFLKEKGGLDGLIYSR
------------------------------------------------------------------

KRQEILDLWVYHTQGYFPDWQNYTPGPGVRYPLTFGWCFKLVPMEPDEVEKATEGENNSLLHPICQ
------------------------------------------------------------------

HGMDDEEREVLIWKFDSRLALKHRAQELHPEFYKDC*
-------------------------------------
```

Supplementary Figure S1 Alignment of amino acid sequences of the HIVconsv and GRIN proteins. GRIN was delivered by non-replicating HAdV-35 and HIVconsv was vectored by plasmid pSG2 DNA and non-replicating poxvirus MVA. GRIN contains full-length proteins of HIV-1 clade A Gag (AY253314), RT, integrase (AF457081) and Nef (AF457081). HIVconsv has 776 amino acids derived from 14 highly conserved regions of HIV-1 proteins among clade consensus sequences as of Los Alamos National Laboratory HIV Sequence Database 2004 (red) as described by Létourneau *at el.* PLoS ONE 2017, of which 604 amino acids (78%) are contained in GRIN (black). Within the 604 amino acids, there are 14 amino acid mismatches (blue), i.e. there was overall 97.6% homology within the regions of HIVconsv between the HIVconsv and GRIN proteins.

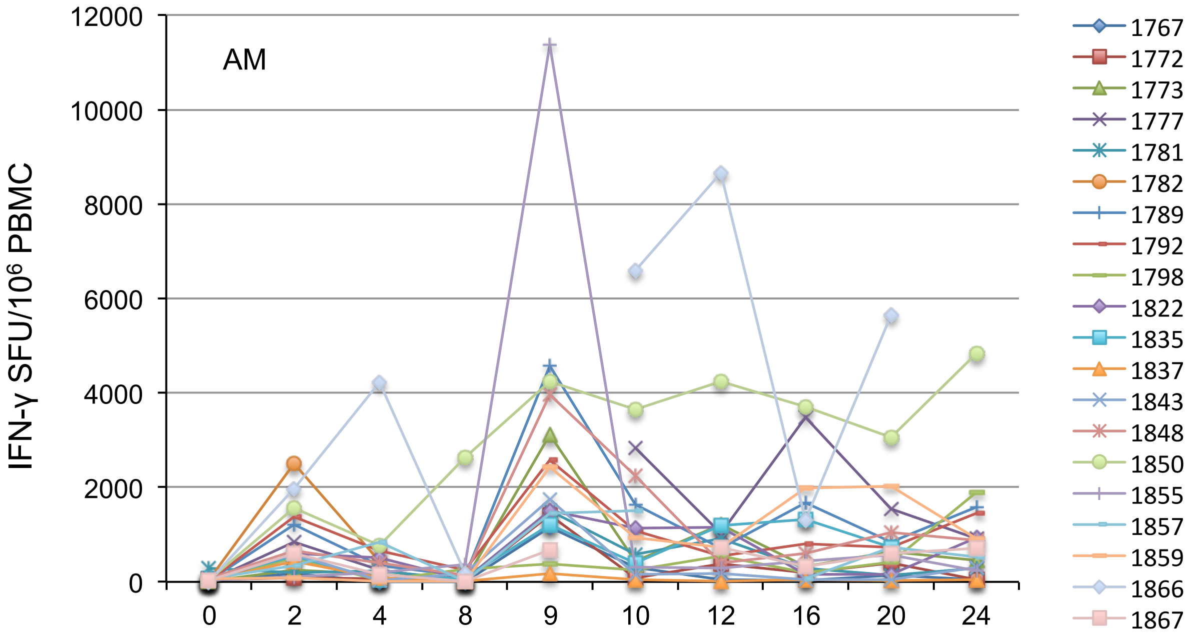

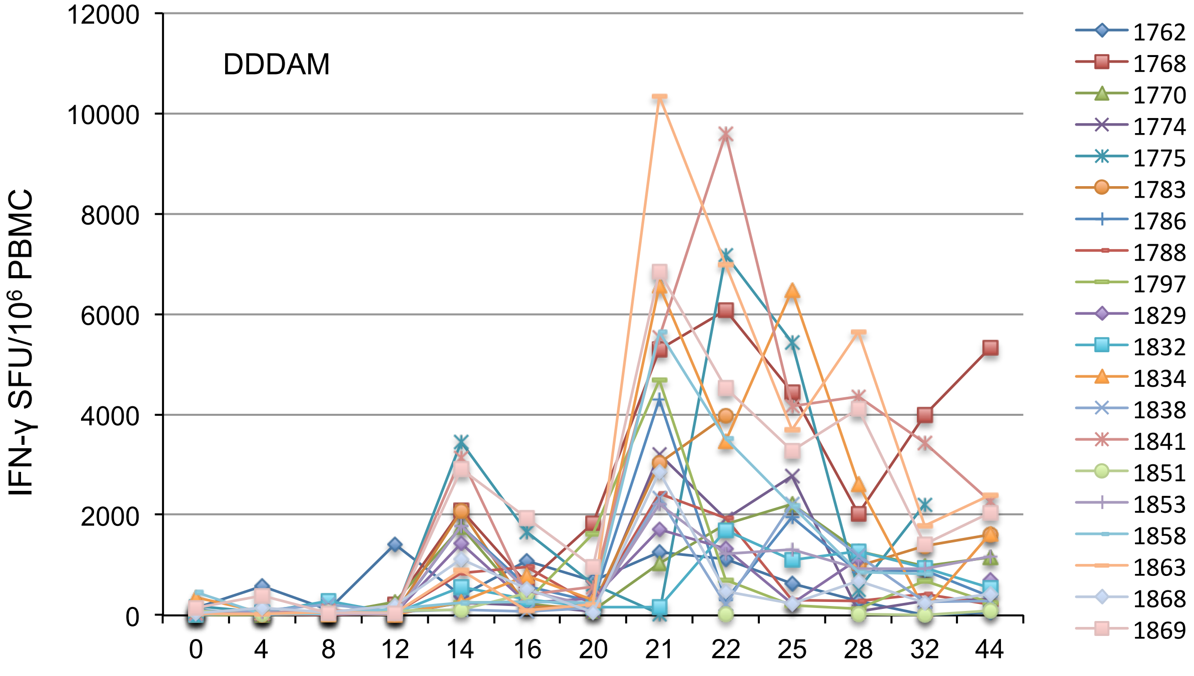

Supplementary Figure S2

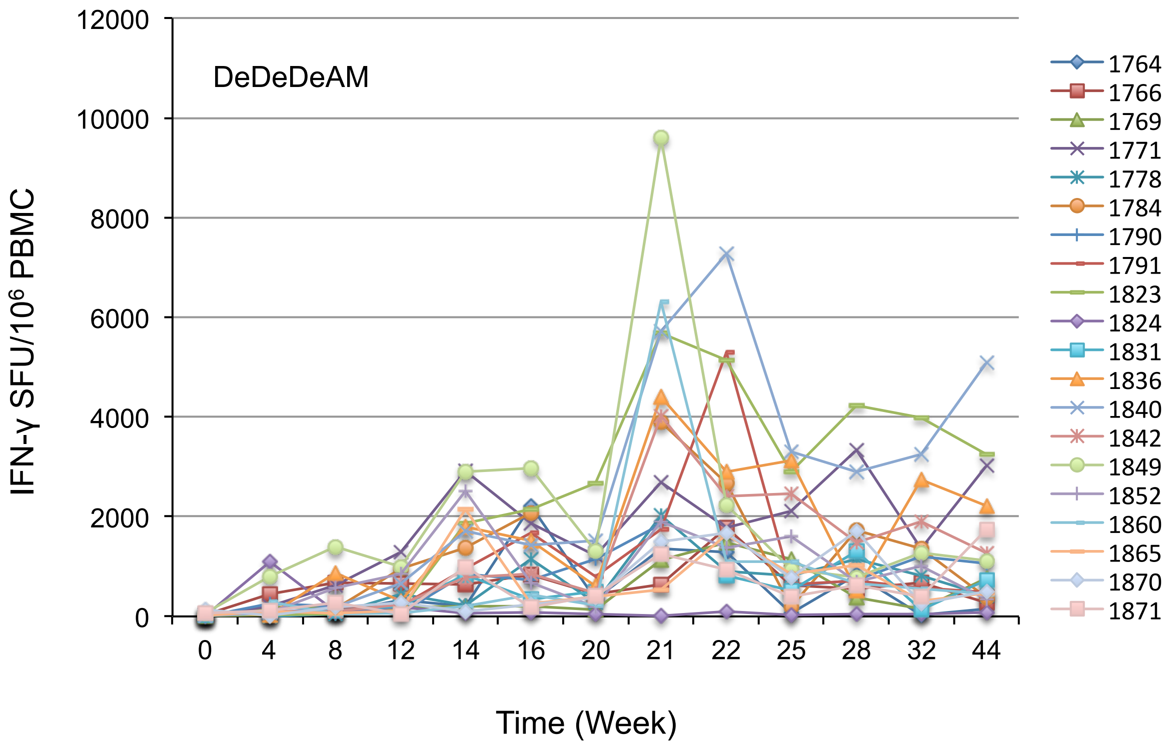

Supplementary Figure S2 Kinetics of the total fresh IFN-γ ELISPOT frequencies of vaccine-elicited T cells shown for individual vaccine recipients. Vaccinations were carried out at weeks 0, 4 and 8 using pSG2.HIVconsv DNA without (D) or with electroporation (De), Ad35-GRIN (A) at week 20 and MVA.HIVconsv (M) at week 25. Placebo responses are not shown.

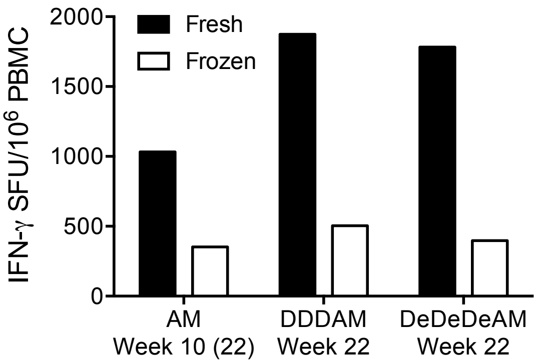

Supplementary Figure S3 Correlation between frequencies determined in an *ex vivo* IFN-γ ELISPOT assay using fresh and frozen PBMC samples. IFN-γ ELISPOT assay on cryopreserved samples was carried out by a reference laboratory (IAVI Human Immunology Laboratory, Imperial College London). The graph compares the frequencies obtained using fresh and frozen samples from 2 weeks after the MVA.HIVconsv administration [for the AM regimen, actual week 10 is shown in **Figure 4a** as week 22], showing approximately 3-fold (AM) and 4-fold (DDDAM and DeDeDeAM) higher frequencies using fresh cells.

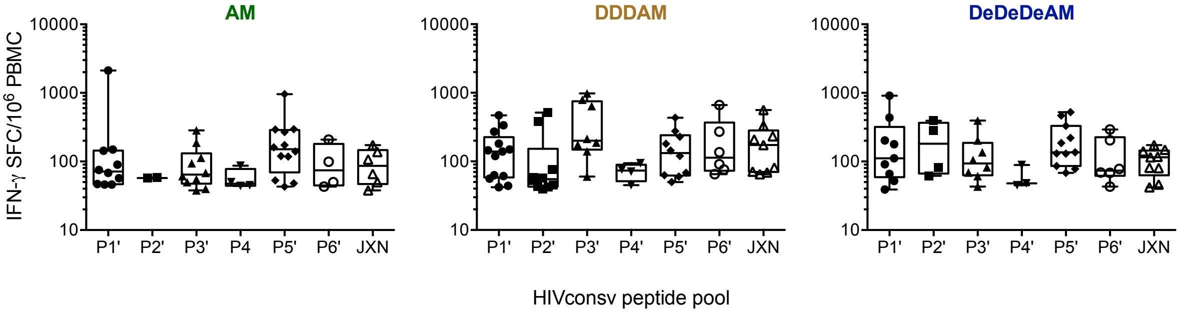

Supplementary Figure S4 Frozen PBMC *ex vivo* IFN-γ ELISPOT assay frequencies of HIVconsv and junctional peptide pool-specific T cells. Apostrophes next to the peptide pools indicate that these pool do not contain peptides spanning regional junction. Thirteen junctional 15-mer peptides are combined in pool JXN contains.

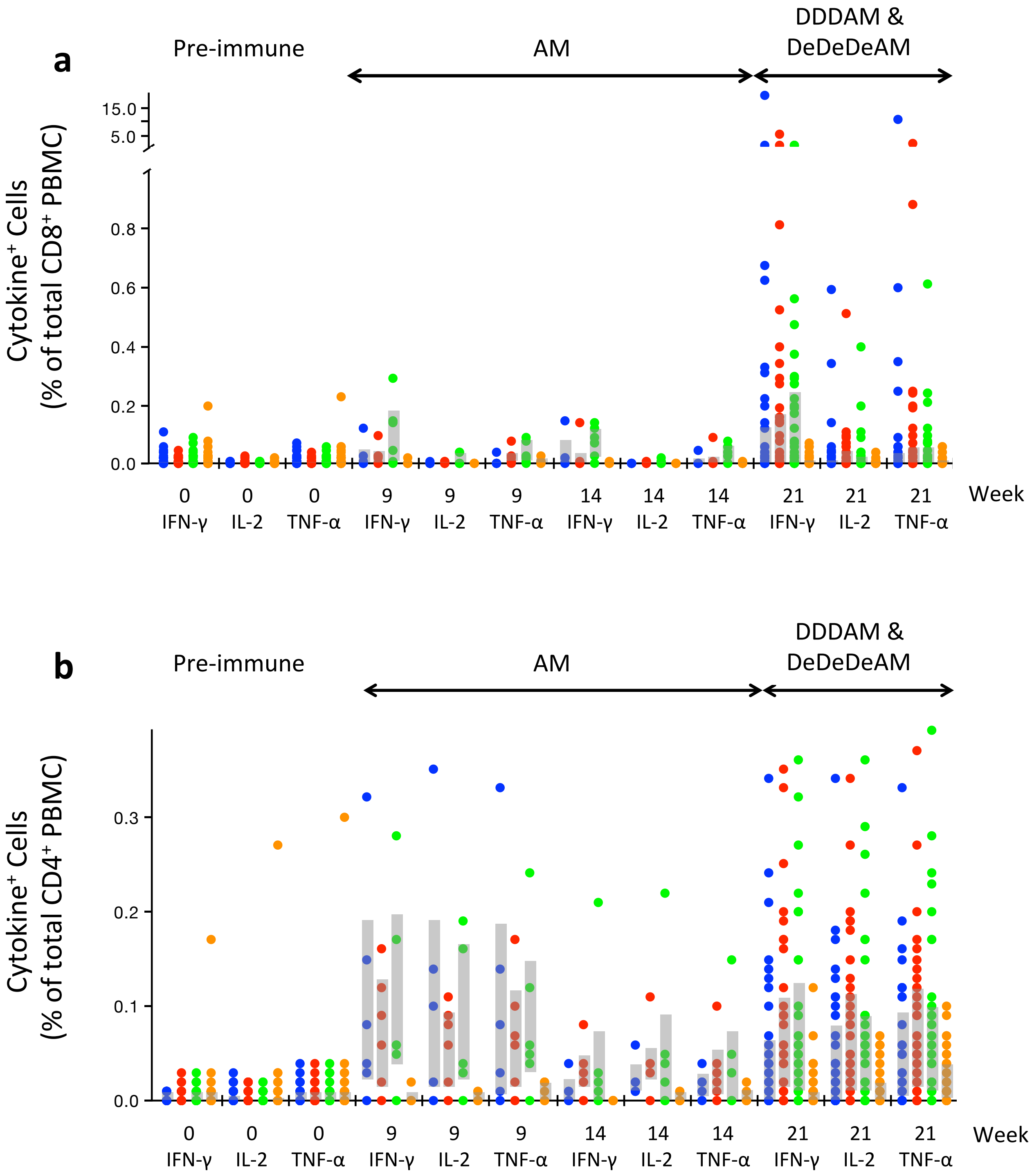

Supplementary Figure S5 Functional analysis of vaccine-elicited T cells. Freshly isolated CD8^{+} (**a**) and CD4^{+} (**b**) PBMC were analyzed before vaccination and 1 week after the last vaccine boost administration for production of IFN-γ, TNF-α and IL-2 cytokines in an ICS assay. Blue – Gag; Red – Pol1; Green – Pol2; Orange – Vif Env.
